# The Timing of Raf/ERK and AKT Activation in Protecting PC12 Cells against Oxidative Stress

**DOI:** 10.1371/journal.pone.0153487

**Published:** 2016-04-15

**Authors:** Qunxiang Ong, Shunling Guo, Liting Duan, Kai Zhang, Eleanor Ann Collier, Bianxiao Cui

**Affiliations:** 1 Department of Chemistry, Stanford University, Stanford, California, 94305, United States of America; 2 Department of Biochemistry, School of Molecular and Cellular Biology, University of Illinois at Urbana-Champaign, Urbana, Illinois, 61801, United States of America; Massachusetts General Hospital/Harvard Medical School, UNITED STATES

## Abstract

Acute brain injuries such as ischemic stroke or traumatic brain injury often cause massive neural death and irreversible brain damage with grave consequences. Previous studies have established that a key participant in the events leading to neural death is the excessive production of reactive oxygen species. Protecting neuronal cells by activating their endogenous defense mechanisms is an attractive treatment strategy for acute brain injuries. In this work, we investigate how the precise timing of the Raf/ERK and the AKT pathway activation affects their protective effects against oxidative stress. For this purpose, we employed optogenetic systems that use light to precisely and reversibly activate either the Raf/ERK or the AKT pathway. We find that preconditioning activation of the Raf/ERK or the AKT pathway immediately before oxidant exposure provides significant protection to cells. Notably, a 15-minute transient activation of the Raf/ERK pathway is able to protect PC12 cells against oxidant strike that is applied 12 hours later, while the transient activation of the AKT pathway fails to protect PC12 cells in such a scenario. On the other hand, if the pathways are activated after the oxidative insult, i.e. postconditioning, the AKT pathway conveys greater protective effect than the Raf/ERK pathway. We find that postconditioning AKT activation has an optimal delay period of 2 hours. When the AKT pathway is activated 30min after the oxidative insult, it exhibits very little protective effect. Therefore, the precise timing of the pathway activation is crucial in determining its protective effect against oxidative injury. The optogenetic platform, with its precise temporal control and its ability to activate specific pathways, is ideal for the mechanistic dissection of intracellular pathways in protection against oxidative stress.

## Introduction

In recent years, preconditioning and postconditioning protection have garnered significant interest as a treatment option for acute brain and heart injuries such as cerebral and cardiac ischemia, and traumatic brain injury[1–3]. Preconditioning refers to the process in which the patient undergoes certain treatment before the injury, such that their brain or heart cells are more resistant to damage when the injury strikes[4]. For example, it has been shown in animals that preconditioning with a brief and mild ischemic episode leads to tolerance against a later, more prolonged and severe ischemic stroke[5,6]. On the other hand, postconditioning refers to the procedure of protecting brain and heart cells from further damage after injury onset [7,8]. Both preconditioning and postconditioning have been demonstrated to reduce myocardial infarction size in diverse species from rodents to pigs and humans[9]. However, significant efforts are needed in order to understand the cellular mechanisms underlying both types of protection, the best time window for treatment and the extent of protection by each treatment[10].

Many studies suggest that pre- and postconditioning achieve cellular protection by activating endogenous antioxidant mechanisms[6,10–12]. During the past three decades, it has been shown that reactive oxygen species (ROS) are involved as key players in acute injuries[13,14]. Generally, acute injuries induce pathophysiological changes that result in the generation of excessive ROS. Overproduction of ROS then leads to cell death by oxidative damage to cellular structures, proteins, lipids and nucleic acids within cells[15,16]. Two well-known pro-survival pathways against oxidative stress, the Raf/MEK/ERK[17] and PI3K/AKT[18,19] pathways, have been shown to be upregulated in both pre- and postconditioning protection [9,20–22]. For example, a recent study has demonstrated early activation of PI3K by addition of PI3K-activating peptide enhances resistance of neurons to hypoxic injury[23].

In order to understand the roles of these signaling pathways in pre- and postconditioning protection, it is crucial to probe the precise timing of specific pathway activation and the resultant cellular protection[24]. Conventional approaches in perturbing intracellular signaling pathways include pharmacological drugs and genetic manipulations. In the former approach, small molecular drugs often have off-target effects in addition to the intended function[23,25,26]. For example, U0126, a widely used inhibitor for the Raf/MEK/ERK signaling pathway, has been recently shown to act as a direct antioxidant[27]. Many drugs bind irreversibly to their targets which prohibit precise control of the treatment duration. On the other hand, the latter approach of genetic manipulation often involves expressing constitutive active mutants or small interference RNAs; thus it is specific to the targeted pathway. However, this approach lacks any temporal control[28]. As such, how preconditioning protection depends on the precise timing of the Raf/ERK or AKT activation is poorly understood.

While traditional genetic approach is limited in temporal control, they could be coupled with emerging optogenetic tools to probe intracellular signaling pathways with excellent specificity and superior temporal resolution[29–32]. Well-designed optogenetic systems have been shown to activate specific signaling components such as Ras [33], beta-catenin [34], phosphoinositides [35] and molecular motors [36]. In particular, two optogenetic systems have been demonstrated to selectively activate the Raf/ERK[37] or AKT[38] pathways by light. In this work, we employed optogenetics to investigate how their protective effect depends on the timings of the pathway activation. PC12 cells, an established neuronal cell line, were used in these studies. For preconditioning, we find that the Raf/ERK pathway is more protective than the AKT pathway and that a 15-minute activation of the Raf/ERK pathway is sufficient to protect against hydrogen peroxide that is added 12 hours later. For postconditioning, the AKT pathway appears to be more protective than the Raf/ERK pathway. Furthermore, the exact timing of postconditioning AKT activation is critical, with an optimal delay of 2 hours from the oxidant exposure to the pathway activation.

## Materials and Methods

### Materials

Hydrogen peroxide (30%, certified ACS) was purchased from Fisher Scientific Ltd. Glucose oxidase, Rose Bengal, rotenone, and paraquat were purchased from Sigma (St. Louis, MO). All oxidative stress inducers except hydrogen peroxide were dissolved in DMSO and stored as frozen stocks at −20°C.

### DNA plasmids

The CIB1-GFP-CAAX plasmid was a gift from Dr. Chandra Tucker in University of Colorado Denver. All other plasmids including CIB1-CAAX, CRY2-mCherry-AKT and CRY2-mCherry-Raf1 were constructed using overlap extension PCR as described in previous literature[37]. In this work, CIB1 refers to a truncated version of CIB1 comprising of amino acids 1–170. CRY2 refers to a truncated version of CRY2 comprising of amino acids 1–498[39]. The AKT domain of the CRY2-mCherry-AKT is a PH domain-truncated variant of AKT.

### Cell culture and transfection

PC12 cells (the neuroscreen-1 sub cell line) (Cellomics, Pittsburgh, PA, USA) [40] were used for cell death assays. NIH 3T3 cells (ATCC) were used for characterizing membrane recruitment of CRY2-mCherry-AKT or CRY2-mCherry-Raf1. For PC12 cells, we used F12K medium (Gibco) supplemented with 15% horse serum (Gibco) and 2.5% fetal bovine serum (FBS) (Gibco). For NIH 3T3 cells, we used DMEM medium (Fisher) supplemented with 10% FBS. All cell cultures were maintained in a standard incubator at 37°C with 5% CO_2_. Transfection was done following manufacturer’s instruction (Fisher Scientific). For one well of a 6-well plate at 80–90% confluency, 1.2 μg DNA was mixed with 3.6 μl Turbofect (Fisher Scientific) in 120 μL serum-free culturing medium and incubated at room temperature for 20 minutes. The DNA/Turbofect mixture was then added to the cell culture drop-wise and incubated for 4 hours before the culture was replenished with 2 ml standard culture medium. Equal molar amounts of constructs were used for transfection of multiple DNA constructs. For Western blot (WB) studies, the electroporation method was used to achieve high transfection efficiency. PC12 cells were grown to 80% confluence in 6-well plates and transferred into a polypropylene tube and centrifuged at 1000 rpm for 3 minutes. The pellet was resuspended in 150 μl of electroporation buffer containing 20μg of DNA in a 4-mm electroporation cuvette. The Amaxa™ Nucleofector™ was used for single-cuvette electroporation. 1 ml of standard culture medium was subsequently added and the mixture was allowed to incubate for 5 minutes before the cells were centrifuged and plated on 6 wells of a 12-well poly-L-lysine-coated cell plate with 1 ml of warm culture medium.

### Western blot

For western blots, 2.0×10^5^ PC12 cells were plated in each well of a PLL-coated 12-well tissue culture plate and transfected with CIB1-GFP-CAAX and CRY2-mCherry-Raf1 or CRY2-mCherry-AKT *via* electroporation. For endogenous pAKT probing, no transfection was performed. After recovering overnight from transfection, cells were starved in serum free medium overnight. The cell culture was then exposed to continuous blue light (0.2 mW/cm^2^) for a fixed amount of time before WB analysis. After light/dark treatment, cells were immediately washed and collected in sample loading buffer (Biorad #1610747), followed by 10 min incubation in 90°C heat block, and chilled on ice immediately after heating. The cell lysate was separated by gel electrophoresis. Protein bands were transferred to a polyvinylidene fluoride membrane (Perkin Elmer) and probed with anti-pAKT(T308)(Cell signaling #9275S), anti-AKT(cell signaling #9272), anti-pERK (Cell signaling #9101S), or anti-ERK antibody (Cell Signaling #9102S). HRP-conjugated secondary antibody was used for protein band detection.

### Differentiation protocol for PC12 cells

PC12 cells were subject to low-serum F12K medium supplemented with 100 ng/ml of nerve growth factor, 1.5% horse serum and 0.25% FBS for 2 days. Then they were washed with PBS for 3 times before the medium was exchanged to standard culture medium and the transfection protocol was performed as described before.

### Cell death assays

Upon the completion of transfection, cells were allowed to recover in standard culture medium for 18 hours before being plated onto 9 wells of a 12-well plate (for every well of transfected cells in a 6-well plate) in low-serum F12K medium supplemented with 1.5% horse serum and 0.25% FBS. This was to ensure that serum activation of the Raf/ERK and AKT pathways was avoided. The plating density was kept at ~150,000 cells per cm^2^. Upon 18–24 hours of dark incubation, the cells were then subjected to various treatments as described. Afterward, propidium iodide (PI) was added to the solution to stain dead cells. The 12-well plate was then scanned under an epifluorescence microscope (Leica DMI6000B microscope) equipped with an automatic scanning stage.

### Addition of different oxidative stressors in PC12 cells

For the H_2_O_2_ treatments, stock solution of H_2_O_2_ (10 mM in water) was diluted to 200 μM with culture medium and added to the culture for a duration of two hours. In the glucose/glucose oxidase assay, 0.01 unit of glucose oxidase was added into glucose-supplemented medium (with one fifth of medium exchanged for high-glucose DMEM) for 24 hours. For the Rose Bengal assay, 1 μM of Rose Bengal was excited at 1 mW/cm^2^ in 1-second 575 nm pulses for every 2 minutes in 1 hour. For paraquat and rotenone assays, 20 mM paraquat or 5 μM rotenone were incubated in the cells for 24 hours respectively. There is no medium replenishment during the oxidative incubation.

### Fluorescence imaging

*Recruitment of CRY2-mCherry-AKT or CRY2-mCherry-Raf1 to membrane studies*: Live cell fluorescence imaging was carried out on an epi-fluorescence microscope equipped with an oil-immersion 100× objective (HCXPL FLUOTAR, N.A. 1.3) and a mercury lamp as the light source. The power density for stimulating light (through a GFP filter cube) was adjusted to ~1 W/cm^2^ at the sample plane. Pulsed light was used for stimulation and imaging.

*Imaging cell death*: Fluorescence imaging for PI stained cultures was carried out on an epi-fluorescence microscope equipped with 10x and 40x objectives and a mercury lamp as the light source. The power density for imaging the cells (through GFP and Texas Red filter cubes) was adjusted to ~0.4 W/cm^2^ at the sample plane. Automatic stage scan was conducted for a defined 7 x 7 grid as detailed in **S1 Fig**.

### Construction of a programmable LED device

For long-term light illumination, a 4-by-3 blue LED array was constructed as described by Zhang et al. [37] 12 blue LEDs were assembled on a breadboard and the light intensity of each LED could be controlled through a tunable resistor. A light diffuser film was positioned above the LED array to make the light intensity homogeneous in the defined area. To avoid cross illumination of different wells, separating barriers were placed around each LED. The light intensity at the cell culture plate was measured by a power meter (Newark, 1931-C).

### Data analysis

*Cell death analysis*: Transfected cells were identified from the GFP channel while dead cells were identified from the Texas Red channel by PI staining, which stains the nuclei and had higher fluorescence intensity than fluorescence from CRY2-mCherry-AKT or CRY2-mCherry-Raf1. The cutoffs are imposed at 2000 arbitrary units for GFP channel and 30000 arbitrary units for the Texas Red channel using imageJ software. Automated CellProfiler software[41] was then used to identify transfected dead cells and transfected live cells through the overlap of images. Transfected cells with stained red nuclei were identified as “dead cells”, while those without stained red nuclei were identified as “live cells”. The percentage of dead cells was computed as “dead cells”/(“live cells” +“dead cells”).

### Statistical analysis

For cell death analysis, the percentage of dead cells in each culture is quantified via automated image analysis. For each condition, each data point is obtained from 3–5 independent sets of experiment. The averaged data is expressed as mean ± standard deviation (SD) unless otherwise specified. Data were analyzed using one-way ANOVA with Dunnett post-hoc test and the results are detailed in **S1 Table**. All figures and statistical analyses were generated using Prism (GraphPad). p < 0.05 was used as a cutoff for statistical significance (*). For p < 0.01, p<0.001 and p<0.0001, the asterisk rating system of **, *** and **** were indicated respectively.

## Results

### Opto-Raf and opto-AKT to activate the Raf/ERK and AKT signaling pathways

**Fig 1:**
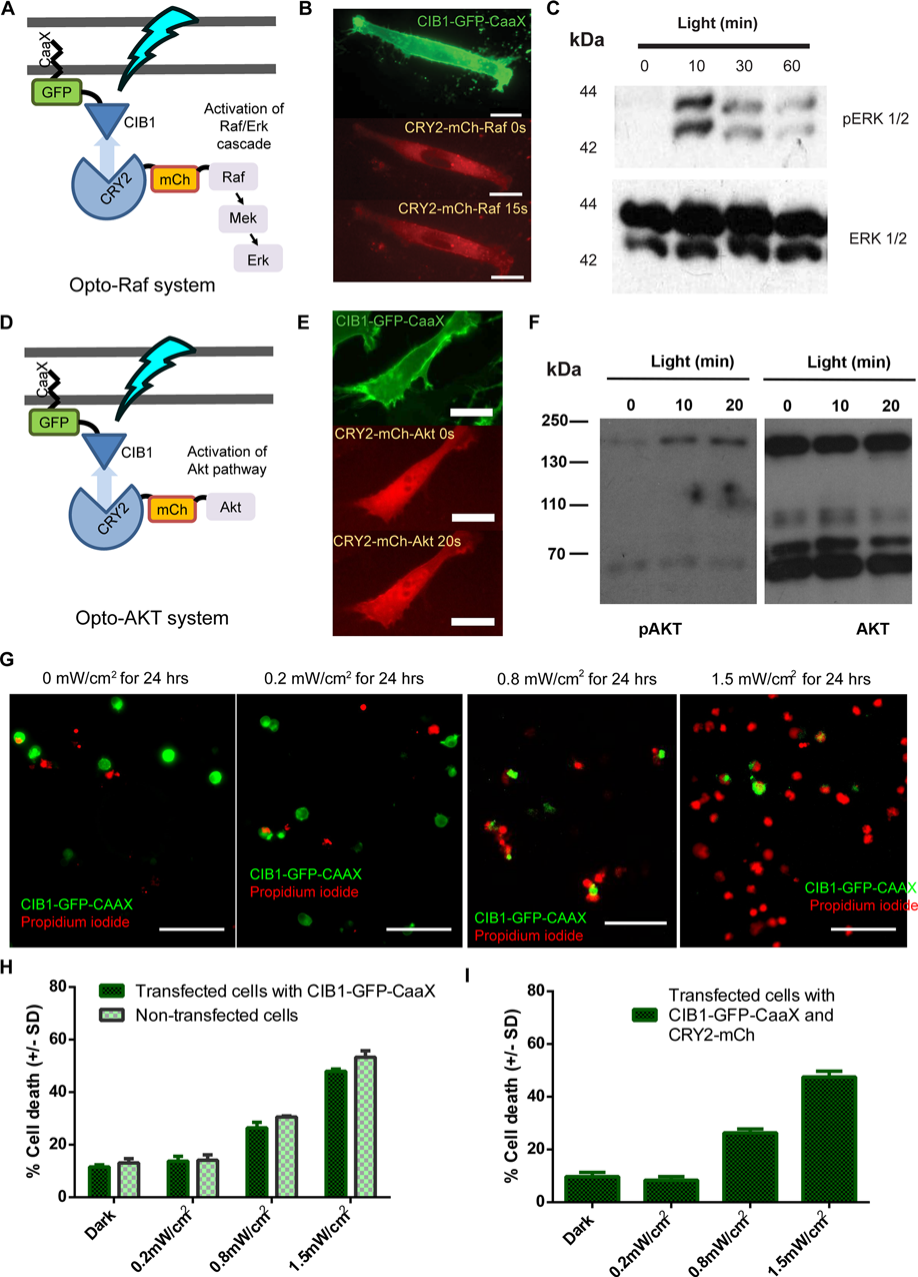
Optogenetic activation of opto-Raf and opto-AKT protects PC12 cells against phototoxicity. (**A**) The design scheme of opto-Raf. The CIB1 domain is anchored to the plasma membrane via a CAAX motif. Upon light stimulation, CIB1-CRY2 interaction should recruit cytoplasmic CRY2-mCherry-Raf1 to the plasma membrane and subsequently activate the downstream ERK pathway. In the dark, CIB1-CRY2 should spontaneously dissociate and return Raf1 to the cytoplasm to inactivate the Raf/ERK pathway. (**B**) CIB1-GFP-CAAX was clearly located at the plasma membrane as indicated by the green fluorescence image. Blue light rapidly recruited cytosolic CRY2-mCherry-Raf1 to the plasma membrane within 15 seconds (**S1 Movie)**. Scale bar = 20 μm. (**C**) Western blot analysis shows phosphor-ERK upon 10, 30 and 60 minutes of blue light activation of opto-Raf. No band was observed when the culture was kept in the dark. (**D**) The design scheme of opto-AKT, similar to that of opto-Raf. Blue light should activate opto-AKT and the pathway. (**E**) Blue light illumination induced rapid recruitment of CRY2-mCherry-AKT from cytosol to the plasma membrane within 20 seconds (**S2 Movie)**. Scale bar = 20 μm. (**F**) Western blot analysis of phosphorylated AKT confirms light-induced activation of CRY2-mCherry-AKT at 160kDa. The endogenous AKT band at 60kDa is not affected by the blue light stimulation. (**G**) Cells transfected with CIB1-GFP-CAAX show phototoxicity at high blue light intensities. Comparable cell death could be seen when the intensities were at 0 and 0.2 mW/cm^2^, while significant cell death was observed when the cells were subjected to 1.5 mW/cm^2^ of blue light. Scale bar = 100 μm. (**H-I**) Quantification of cell death rates for cells transfected with CIB1-GFP-CAAX only (**H**) or CIB1-GFP-CAAX and CRY2-mCherry (**I**) that were exposed to blue light at 0, 0.2, 0.8 and 1.5 mW/cm^2^ for 24 hours. Untransfected cells in the same culture were used as controls. Each set of data comprises of 5 sets of experiments with 2000–5000 cells each. Data is represented as mean +/- standard deviation.

Our optogenetic control of intracellular signaling pathways is based on light-induced dimerization of cryptochrome 2 (CRY2) and its interacting partner CIB1. As illustrated in **Fig 1A**, the opto-Raf system is consisted of two engineered proteins CIB1-GFP-CAAX and CRY2-mCherry-Raf1. In the dark, the CIB1-GFP-CAAX protein is tethered to the plasma membrane *via* its CAAX motif, while the CRY2-mCherry-Raf1 is diffusive in the cytosol. Upon blue light stimulation, conformational change in CRY2 engages its binding to CIB1 and thus recruits CRY2-mCherry-Raf1 to the plasma membrane. This is confirmed by fluorescence imaging as shown in **Fig 1B** and **S1 Movie**, where CRY2-mCherry-Raf1 was recruited to the plasma membrane within 15 seconds of blue light illumination. Many kinases, including Raf and AKT, are known to be spontaneously activated when recruited to the membrane[42,43]. Indeed, western blot analysis of phosphor-ERK confirmed the activation of the Raf/ERK pathway after 10, 30 and 60 minutes of blue light illumination (**Fig 1C**). The design of the opto-AKT system is similar to that of the opto-Raf system. It consists of two engineered proteins CIB1-GFP-CAAX and CRY2-mCherry-AKT (**Fig 1D**). Membrane recruitment of CRY2-mCherry-AKT is visible after 20 seconds of blue light illumination (**Fig 1E** and **S2 Movie)**. Western blot analysis of phosphor-AKT in **Fig 1F** confirmed the activation of the CRY2-mCherry-AKT (the 160kDa band) after 10 and 20 minutes of blue light illumination. The native AKT (the 60kDa band) was not affected by the blue light stimulation.

### Optimization of the light intensity for cell protection studies

In order to utilize optogenetic systems to study effects of pathway activation on cell protection, it is crucial to ensure that phototoxicity is minimal, especially for experiments that require long periods of monitoring. We first probed blue-light induced phototoxicity in non-transfected PC12 cells and in PC12 cells expressing CIB1-GFP-CAAX alone. For this study, blue light illumination was carried out using a custom-built LED light box housed inside a CO_2_ incubator. Upon 24 hours of continuous exposure to blue light at 0, 0.2, 0.8 or 1.5 mW/cm^2^, the dead cells were identified *via* PI staining. Each culture well was automatically scanned and the cell death rate in each culture was then quantified through automated data analysis (**S1 Fig**). Representative images in **Fig 1G** show that the number of PI-stained dead cells increased as the light intensity was raised from 0 to 1.5 mW/cm^2^. Measurements of 3 experimental sets (2000–5000 cells for each experiment) indicate that PC12 cells exposed to 0.2 mW/cm^2^ blue light exhibit comparable death rate to those kept in dark, for both non-transfected and CIB1-GFP-CAAX transfected cells (**Fig 1H**). Higher light intensities at 0.8 mW/cm^2^ and 1.5 mW/cm^2^ induced higher cell death rates. This result corroborates our previous observations[37]. Thus, the light intensity for subsequent experiments was kept at 0.2 mW/cm^2^, a level at which 24 hours of continuous exposure did not cause detectable phototoxicity. As another control, we transfected PC12 cells with CIB1-GFP-CAAX and CRY2-mCherry. The CRY2-mCherry plasmid was based on the similar vector as that of CRY2-mCherry-Raf1 or CRY2-mCherry-AKT. After blue light exposure for 24 hours at different intensities, the death rates were measured by PI staining. The death rates and the trends are similar to that of CIB1-GFP-CAAX singly transfected cells, indicating that the light-induced CRY2-CIB1 interaction or the expression of CRY2 does not affect cell health (**Fig 1I**).

We noticed that optogenetic activation of the Raf/ERK and PI3K/AKT pathways were able to protect PC12 cells against phototoxicity induced by high intensities of blue light. We observed that the opto-Raf activation completely eliminated blue light-induced phototoxicity at intensities of 0.8 and 1.5 mW/cm^2^ (**S2A Fig**). The opto-AKT activation also protected transfected cells against phototoxicity, but the protective effect was significantly less than that of opto-Raf (**S2B Fig**).

### Light-induced activation of opto-Raf and opto-AKT protects PC12 cells against hydrogen peroxide

**Fig 2:**
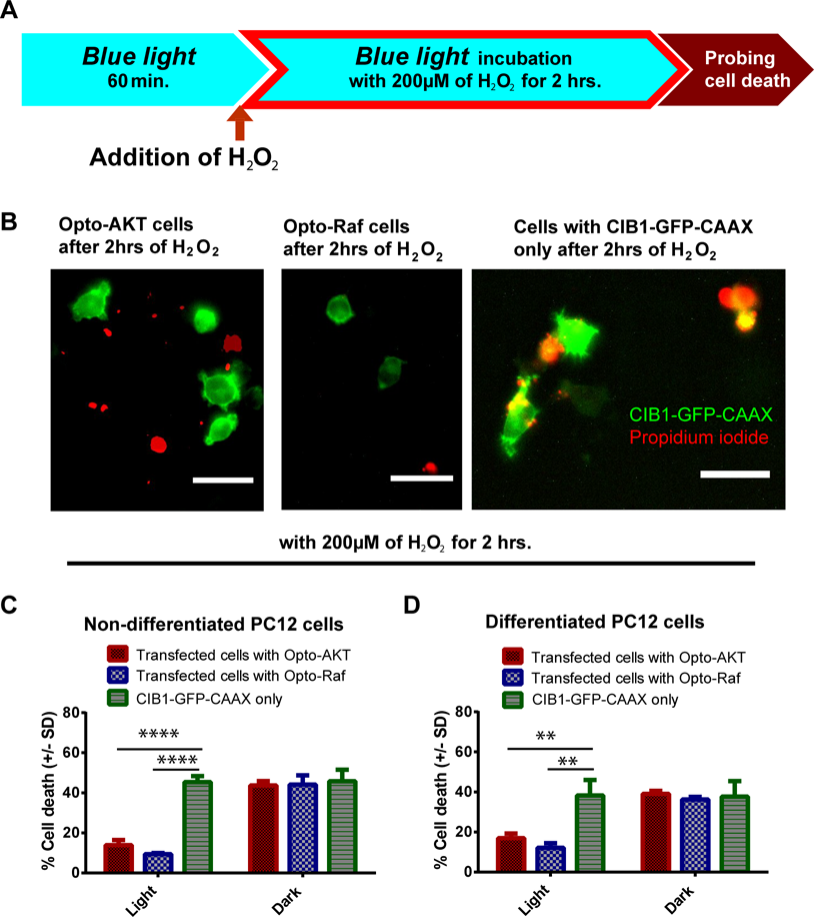
The activation of opto-Raf or opto-AKT protects undifferentiated and differentiated PC12 cells against hydrogen peroxide. (**A**) Transfected PC-12 cells with CIB1-GFP-CAAX only, opto-AKT, or opto-Raf were first illuminated with 0.2 mW/cm^2^ blue light for 1 hour before being subjected to 200 μM of hydrogen peroxide. The blue light (480 nm) was kept on throughout the duration of the experiment. Cell death was probed *via* PI staining. (**B**) Undifferentiated PC12 cells transfected with opto-Raf or opto-AKT demonstrated less cell death after they were exposed to 200 μM of hydrogen peroxide for 2 hours. The red channel was thresholded (cut-off at 30,000 arbitrary units) to represent PI-stained dead cells. Scale bar = 50 μm. (**C**) Hydrogen peroxide treatment to undifferentiated PC12 cells at 200 μM for 2 hours showed that the activation of opto-AKT and opto-Raf exerted a prominent protective effect compared to CIB1-GFP-CAAX control. (**D**) Hydrogen peroxide treatment to differentiated PC12 cells at 200 μM for 2 hours showed that the activation of opto-AKT and opto-Raf exerted a prominent protective effect compared to CIB1-GFP-CAAX control. In (**C**) and (**D**), the dark controls showed comparable cell death to CIB1-GFP-CAAX singly transfected controls. For all the results, each set of data comprises of 3 sets of experiments with 1000–3000 cells each. Data is represented as mean +/- standard deviation.

We confirmed that opto-Raf and opto-AKT are effective in protecting PC12 cells against H_2_O_2_. H_2_O_2_ is one of the most widely used oxidative stressors for experimental studies due to its central role in oxidative damage in cells[44]. We first activated opto-Raf and opto-AKT for 1 hour to ensure that the pathway was already activated before stressor exposure. Then, PC12 cells were subjected to 200 μM H_2_O_2_ for 2 hours while the cells were subjected to continuous blue light stimulation during the entire treatment period (**Fig 2A**). After treatment, cell death rates were measured by PI staining.

Our results indicate that the light-induced activation of Raf/ERK and AKT pathways consistently protects PC12 cells against 200 μM H_2_O_2_. Fluorescence images in **Fig 2B** show that H_2_O_2_ treatment resulted in less cell death in PC12 cells transfected with opto-Raf and opto-AKT (double colored cells indicate transfected dead cells) as compared with those transfected with CIB1-GFP-CAAX only. Quantification of the cell death rates for PC12 cells showed that opto-Raf and opto-AKT activated under blue light conferred protective effects with much lower death rates at 13.9% and 9.5% respectively as compared to CIB1-GFP-CAAX control at 45.4% (**Fig 2C**).

We also verified that opto-Raf and opto-AKT are protective to NGF-differentiated PC12 cells as well as NIH 3T3 cells. NGF-differentiated PC12 cells show long neurites extending from the cell bodies (**S3 Fig** and see Methods). Similar to undifferentiated PC12 cells, blue light activation of opto-Raf and opto-AKT pathways are highly protective to NGF-differentiated PC12 cells, bringing down the cell death rate to 16.9% and 12.1% respectively as compared with 40% cell death for control cells (**Fig 2D**). We further demonstrated that the phenomenon of protection rendered by opto-Raf and opto-AKT is also not restricted to PC12 cells. For 3T3 cells, the cell death rates were also significantly reduced when opto-Raf and opto-AKT were activated, (**S4 Fig**).

### Light-induced activation of opto-Raf and opto-AKT protects PC12 cells against various types of oxidative stress

**Fig 3:**
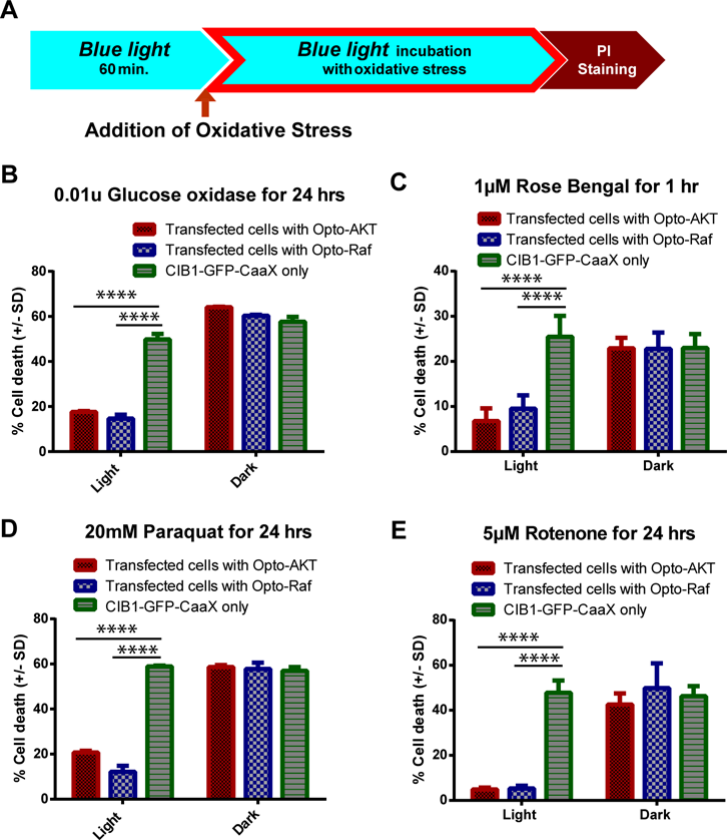
The activation of opto-Raf or opto-AKT protects PC12 cells against different types of oxidative stressors. (**A**) Opto-Raf and opto-AKT activation protected the cells against continuous ROS production from glucose oxidase. (**B**) Opto-Raf and opto-AKT activation protected the cells against singlet oxygen species produced by Rose Bengal upon green light excitation. (**C**) Opto-Raf and opto-AKT activation protected cells against 20 mM paraquat treatment for 24 hours. (**D**) Opto-Raf and opto-AKT protected cells against 5 μM rotenone incubation for 24 hours. In (**A**) to (**D**), the dark controls showed comparable cell death to CIB1-GFP-CAAX singly transfected controls. For all the results, each set of data comprises of 3–5 sets of experiments with 2000–3000 cells each. Data is represented as mean +/- standard deviation.

In addition to H_2_O_2_, we have tested the protection effect of opto-Raf and opto-AKT against four other types of commonly used oxidative stressors including glucose oxidase, Rose Bengal, rotenone, and paraquat (**Fig 3A**). Glucose oxidase, in the presence of glucose, produces a steady release of hydrogen peroxide and has been used in several oxidative stress studies[45]. Rose Bengal is a photosensitizer that produces singlet oxygen species upon green light illumination[46,47]. Rotenone induces a variety of oxidative radicals, especially hydroxyl radicals, by blocking the electron transport chain in mitochondria[48]. Paraquat generates mostly superoxide free radicals in cells[49]. The concentrations and the treatment durations of each oxidant are as reported in previous literatures and indicated in **Fig 3B–3E**.

With blue light stimulation, both opto-Raf and opto-AKT drastically reduced the cell death rates for all these oxidative stressors. In the dark, PC12 cells transfected with opto-Raf, opto-AKT, or CIB1-GFP-CAAX only, show similar cell death rates to oxidant treatments. For light exposure, blue light was turned on for 60min before adding the oxidative stressor and was kept on during the entire treatment (**Fig 3A**). Data analysis shows that the death rates of cells transfected with opto-Raf or opto-AKT are much lower upon blue light stimulation (**Fig 3B–3E**), indicating that opto-Raf and opto-AKT are protective against general ROS damage.

### Preconditioning activation of the Raf/ERK or AKT signaling pathways protect PC12 cells against a subsequent oxidative insult

**Fig 4:**
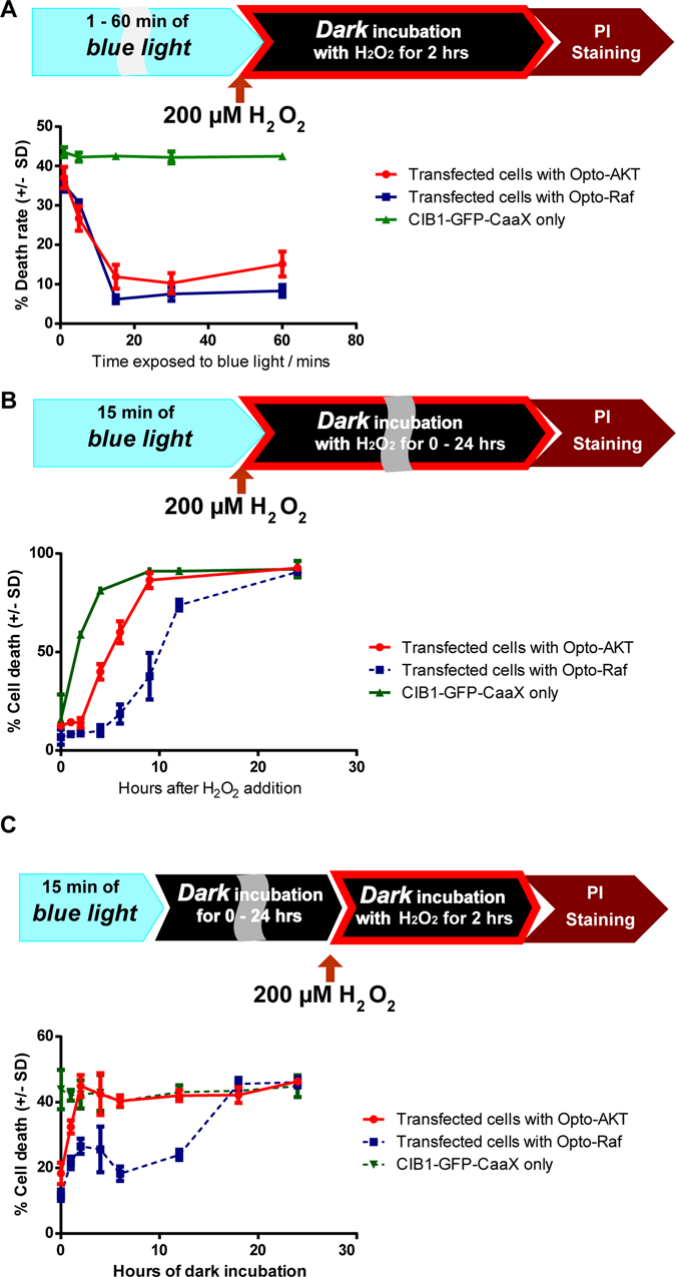
**The timings of preconditioning activation of opto-Raf and opto-AKT in cell protection** (**A**) Varied duration of blue light (1, 5, 15, 30, or 60 minutes) was provided to the cells before they were incubated with 200 μM of hydrogen peroxide under dark conditions for 2 hours. The results indicate that only 15 minutes of prior activation was required to achieve maximum preconditioning effects for both opto-Raf and opto-AKT systems. (**B**) PC12 cells were illuminated with blue light for 15 minutes before they were incubated with 200 μM of hydrogen peroxide for a variable duration. The cells were kept in dark during hydrogen peroxide incubation. It was found that opto-Akt activation provided full protection for 2 hours while opto-Raf activation provided full protection for 6 hours. (**C**) PC12 cells were illuminated with blue light for 15 minutes before they were placed in dark for varied hours (termed as buffer period). Then, 200 μM of hydrogen peroxide was added to the culture and incubated for 2 hours under dark. Preconditioning activation of opto-Raf exhibited a delayed protective phase even after 12 hours of buffer period, while opto-AKT completely lost its protective effects after 2 hours of buffer period. For all the results, each set of data comprises of 3–5 sets of experiments with 1000–3000 cells each. Data is represented as mean +/- standard deviation.

For preconditioning studies, we first examined whether transient activation of the Raf/ERK or AKT pathways before the oxidative insult exerted a protective effect on the cells. In this study, blue light was turned on for 1, 5, 15, 30, or 60 min to activate Raf/ERK or AKT pathways in undifferentiated PC12 cells. Then, blue light was turned off and the cultures were treated with 200 μM of hydrogen peroxide for 2 hours under darkness, followed by PI staining for death rate analysis. Our results indicate that preconditioning activation for 15 minutes or longer resulted in cell death rates at 6–8% for opto-Raf and 11–15% for opto-AKT. These death rates are drastically lower as compared to their respective dark controls at ~42% (**Fig 4A**). Blue light stimulation for 5 minutes also resulted in lower cell death rates (31% for opto-Raf and 26% for opto-AKT) than the dark controls, but the protective effect is not optimal. Hence, preconditioning activation of the Raf/ERK or AKT pathway for 15 minutes or longer has a powerful protective effect against oxidative stressors that are applied later.

Next, we probed the extent of the protection provided by a 15-minute preconditioning activation of opto-Raf or opto-AKT. For this set of studies, blue light was turned on for 15 minutes to activate either opto-Raf or opto-AKT. Then, the cultures were treated with 200 μM of H_2_O_2_ for a variable duration of 0, 1, 2, 4, 6, 9, 12, 16, or 24 hours, followed by immediate PI-staining for dead cells. During the H_2_O_2_ treatment period, the cultures were maintained in darkness. **Fig 4B** (red curve) demonstrates that a 15-minute activation of opto-AKT led to a significant protective effect for the first 2 hours, with death rates (~14% at 2 hours) comparable to that of cultures not treated with H_2_O_2_ (~12% at 0 hour) and significantly lower than that for the CIB1-GFP-CAAX control culture (~59% at 2 hours). However, the cell death rates increased dramatically at the 2^nd^ hour to 9^th^ hour window and eventually matched the cell death rate of the control culture at the 9^th^ hour mark. Conversely, a preconditioning 15-minute activation of opto-Raf achieved a much longer protection (blue curve in **Fig 4B**). The cell death rate remained low for the first 6 hours (18.6% at the 6^th^ hour), and gradually increased over the 6^th^ to 24^th^ hour window.

In addition to undifferentiated PC12 cells, we also probed the extent of the protection provided by a 15-minute preconditioning activation of opto-Raf or opto-AKT in differentiated PC12 cells. Similar to undifferentiated PC12 cells, NGF-differentiated PC12 cells transfected with opto-Raf (blue curve) or opto-AKT (red curve) had significant lower death rates compared to CIB1-GFP-CAAX control culture (**S5A Fig**). Also consistent with undifferentiated PC12 cells, opto-Raf has much longer duration of protection than opto-AKT (**S5A Fig**).

### Preconditioning protection depends on the timing of the pathway activation before the oxidative insult occurs

The time course of preconditioning protection has generally been classified in two phases: a rapid phase where the trigger induces protection within minutes and a delayed phase where the protected state develops over hours and requires *de novo* protein synthesis[4]. In the previous section where opto-Raf or opto-AKT was activated immediately before adding H_2_O_2_, we probed the rapid phase of preconditioning. Here, we examined whether the delayed phase of preconditioning protection exists in either of the pathways. For this study, undifferentiated PC12 cells expressing either opto-Raf or opto-AKT were stimulated with blue light for 15 minutes. Then, the cells were kept in dark for a variable period of 0 to 24 hours (referred as the buffer period) before being exposed to 200 μM H_2_O_2_ for 2 hours (**Fig 4C**). The cells were subsequently stained with PI for cell death analysis.

**Fig 4C** shows that a 2-hour buffer period between the activation of opto-AKT and oxidant exposure completely eliminated its preconditioning protective effect. In contrast, the preconditioning activation of opto-Raf for 15 minutes showed significant protective effect even after a buffer period of 12 hours. We had also performed the same set of experiments for differentiated PC12 cells and observed similar protective effect from opto-Raf activation (**S5B Fig**). These results suggest that preconditioning activation of opto-Raf comprises both rapid and delayed phases of protection, while preconditioning activation of opto-AKT comprises of only the rapid phase. The timing difference is not due to different rates in ERK and AKT dephosphorylation, as it has been previously demonstrated *via* western blot that both the phosphor-ERK and phosphor-AKT returned to basal levels within an hour[37,50]. We also carried out a western blot analysis of phosphor-ERK that showed negligible phosphor-ERK at the 2^nd^ and 6^th^ hour mark after 15 minutes of opto-Raf stimulation (**S6 Fig**).

A notable feature of opto-Raf (**Fig 4C**, blue curve) is a peak in the death rate at the 2-hour buffer period. After 15-minute preconditioning activation of Raf/ERK, the cell death rate with a 6-hour buffer period is lower than that with a 2-hour buffer period. We repeated these experiments for five independent times, and the peak at the 2^nd^ hour mark is noted each time (**S7 Fig**). The unique feature of the cell death profile clearly indicates that the protective effect conveyed by a 15-minute opto-Raf activation is not a graded phenomenon. Rather, it depends strongly on the time interval between the pathway activation and the oxidative strike.

### Postconditioning activation of the AKT pathway is more protective than the Raf/ERK pathway against oxidative stress

**Fig 5:**
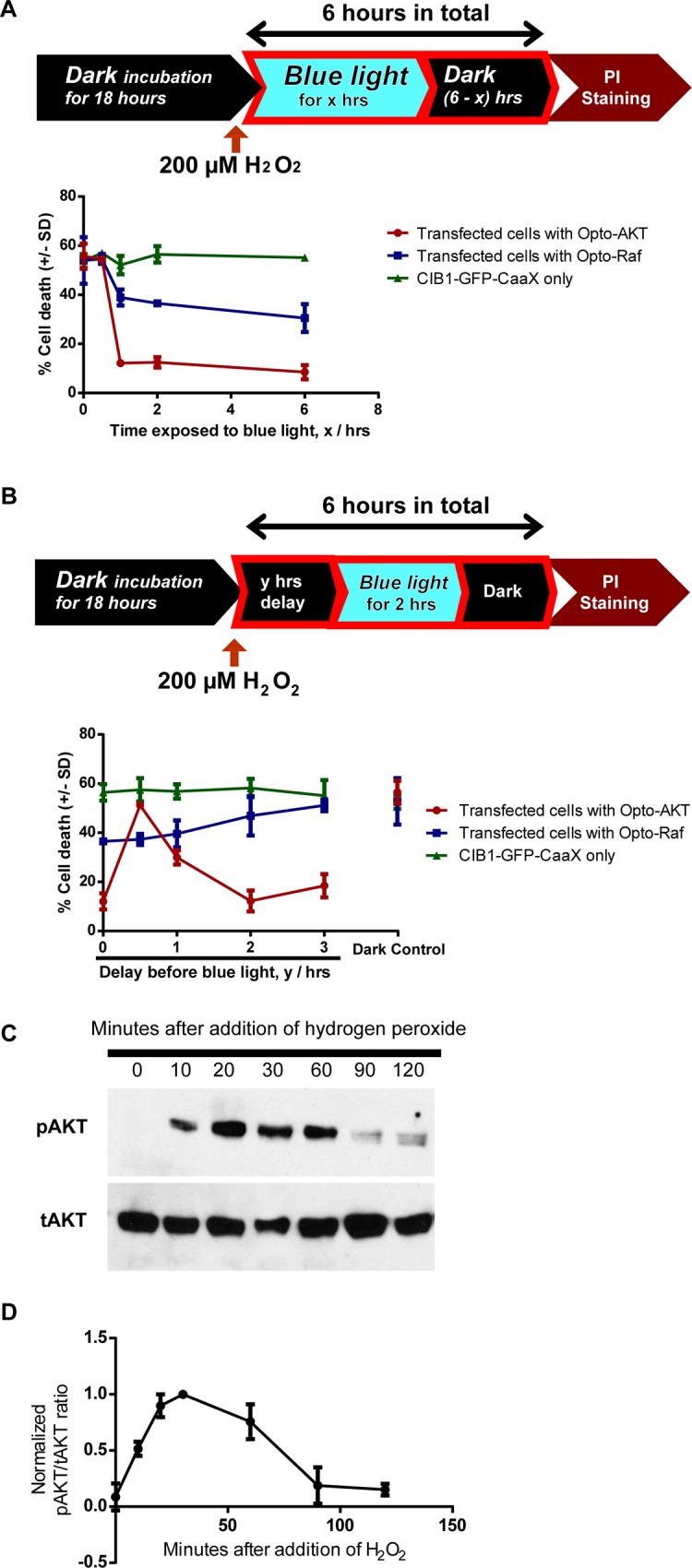
**The timings of postconditioning activation of opto-Raf and opto-AKT in cell protection** (**A**) PC12 cells were kept in dark for 18 hours before the addition of 200 μM hydrogen peroxide. The cells were incubated with hydrogen peroxide for a total of 6 hours, during which the cells were illuminated with blue light for a variable period. PI staining was used to probe cell death rates. The results show opto-AKT is significantly more protective than opto-Raf in postconditioning activation of signaling pathways. (**B**) PC12 cells were kept in dark for 18 hours before they were incubated with 200 μM hydrogen peroxide for 6 hours. 2 hours of blue light illumination (480 nm at 0.2 mW/cm^2^) was started after 0, 0.5, 1, 2, or 3 hours of delay. Opto-Raf cells showed a gradual increase in cell death as the delay increased. In contrast, opto-AKT cells showed maximal protection when the delay time was 2 hours. A delay time of 30min resulted in no protection at all. Each set of data in (**A**) and (**B**) comprises of 3–5 sets of experiments with 2000–3000 cells each. Data is represented as mean +/- standard deviation. (**C**) and (**D**) Western blot analysis of phosphorylated AKT against total AKT shows a peak of pAKT phosphorylation at 30 minutes and 4 hours after encountering 200 μM hydrogen peroxide.

Building upon our observation that the Raf/ERK pathway is more effective than the AKT pathway in the preconditioning protection, we explored their relative effectiveness in postconditioning protection. For postconditioning protection, the pathways were activated after the cells were exposed to oxidative stressors[51]. The cultures were first maintained in dark for 18 hours then subjected to 200 μM H_2_O_2_ treatment for a total of 6 hours. Immediately after the H_2_O_2_ addition, blue light was turned on for 0, 0.5, 1, 2 or 6 hours to activate either opto-Raf or opto-AKT. After 6 hours of H_2_O_2_ exposure, the cell death rate of each culture was probed via PI staining. The results (**Fig 5A**) indicate that 1-hour or longer activation of opto-AKT drastically reduced the cell death rate (~12%) as compared with the control cultures (~56% for dark control and ~52% for CIB1-GFP-CAAX control). The activation of opto-Raf for 1 hour or longer also resulted in reduced cell death rates as compared with the controls, but the death rates were much higher than those with AKT activation at the same conditions (~39% death rate).

### The precise timing of opto-AKT is critical for its postconditioning protection

Finally, we explored whether a delayed activation of the Raf/ERK or AKT pathway could still protect cells under oxidative stress. For this purpose, blue light was turned on after 0, 0.5, 1, 2 or 3 hours of delay period following the addition of H_2_O_2_. The duration of the blue light illumination was fixed at 2 hours (**Fig 5B**). For opto-Raf, delayed activation from 0 to 3 hours led to diminished protective effect with increased cell death rates. Interestingly, for opto-AKT, a 30-minute delay period almost completely abolished the protective effect. However, there appeared to be an optimal delay period of 2 hours, which displays similar protective effects as opto-AKT activation without any delay (**Fig 5B**). Five sets of independent experiments show consistent results that a 30min-delay almost abolishes the protective effect of opto-AKT while a 2hr-delay recovers the protective effect (**S8 Fig**). To further confirm this result, we reduced the duration of blue light exposure to 1 hour, while the delay period was set at 0, 0.5, 1 or 2 hours. This set of experiments show similar results–a maximum death rate for a delay period of 30 min (**S9 Fig**).

In order to understand the high death rate for opto-AKT postconditioning with 30-min delay, we carried out the western blot analysis of endogenous AKT activities at 0, 10, 20, 30, 60, 90, and 120min after H_2_O_2_ addition. For this study, the cells were not transfected and the endogenous AKT and pAKT were probed. As shown in **Fig 5C and 5D**, H_2_O_2_ treatment elicited pAKT activation that peaks around 30min and gradually decreases afterward. At 90 and 120min after H_2_O_2_ addition, the pAKT levels are very low. H_2_O_2_-induced AKT activation has been reported before [52]. We hypothesize that the high level of endogenous pAKT at 30min renders opto-AKT less useful as the factors downstream of AKT are already activated. As the endogenous pAKT level decays at longer delay time, the protective effect of opto-AKT activation became obvious. This set of results emphasizes that, for therapeutic purpose, the timing of signaling pathway activation is highly critical for postconditioning treatment.

## Conclusions and Discussion

We employed optogenetic approaches to investigate the timing of pre- and postconditioning protection by the Raf/ERK and AKT pathways against oxidative stresses. A key theme explored in this study is to define the temporal regimes at which the pro-survival pathways are protective. The timing between the pathway activation and the oxidative insult has been shown to be important for preconditioning protection[4,6,10]. For preconditioning, we observed that a transient 15-minute activation of the Raf/ERK pathway elicited a prolonged protection for oxidative strike that were applied many hours later, while the protective effect of the AKT pathway was completely wiped out by two hours of delay time. For postconditioning, however, the AKT pathway was more protective than the Raf/ERK pathway.

Our measurements have led to interesting findings that there exists optimal delay time for both the Raf/ERK pathway in preconditioning studies and for the AKT pathway in postconditioning studies. The existence of an optimal time window for preconditioning protection has been indicated in some previous studies. For example, the optimal delay time for cardiac preconditioning is between 5 minutes and 60 minutes[53]. If the delay time is either less than 1 minute or longer than a few hours, there is minimal protection[54][55]. The existence of an optimal time window for postconditioning protection has not yet been reported. Our results indicate that, the exact treatment timing following injury should be carefully examined in order to achieve the best results.

The differential protective effects of Raf/ERK and AKT pathways in pre- and post-conditioning could be attributed to their distinct protection mechanisms. The PI3K/AKT pathway is known to transduce its pro-survival signals via phosphorylation-dependent suppression of apoptotic factors like BAD, forkheard transcription factor (FOXO) and IKKɑ [56–59]. On the other hand, the Raf/ERK pathway has been largely shown to activate transcription factors such as Nrf-2 and CREB[60], which are known to upregulate the synthesis of new antioxidant proteins. New protein synthesis downstream is likely contributing to the 12-hr protection period following a 15min blue light stimulation of opto-ERK in pre-conditioning. Conversely, in postconditioning, the immediate phosphorylation of apoptotic partners by AKT may be more critical in ensuring the survival of cells.

In conclusion, optogenetics offers the ability to activate intracellular signaling pathways with specificity and temporal precision, which make it possible to investigate the critical timings for preconditioning or postconditioning protection. We envision that similar approach could be extended to other signaling pathways involved in preconditioning such as PKCɛ pathway[61].

## Supporting Information

S1 Fig Optimization of automatic scanning and threshold cutoffs. **(A)** Stage scan of an entire 12-well plate. 7 x 7 images were captured within the 10 x 10 area outlined for each data point. **(B)** Maximum fluorescence intensity of CRY2-mCherry-Akt protein was typically below 5,000, while maximum fluorescence intensity of propidium iodide was at around 60,000. This formed the basis of allowing thresholding levels, where the cut-off was imposed at 30,000. Similar intensities of light were used when capturing the images on the epifluorescence microscope for consistency. **(C)** The application of thresholding allowed us to differentiate between transfected dead cells and transfected cells that are alive. In this example, CRY2-mCherry-Akt and CIB1-GFP-CAAX were transfected in the cells. Scale bar = 50 μm.
(JPG)

S2 Fig Controls transfected with CIB1-GFP-CAAX and CRY2-mCherry only showed no protective effects against blue light-induced phototoxicity. Cells were transfected with CIB1-GFP-CAAX and CRY2-mCherry, and placed under 0, 0.2, 0.8 and 1.5 mW/cm^2^ of blue light illumination for 24 hrs. Percentage of cell death was then probed via propidium iodide staining. The cell death rates shown in Fig 2 are comparable to the ones transfected singly with CIB1-GFP-CAAX, showing that CRY2-mCherry fragments do not affect the cell death rates significantly with/without blue light.
(JPG)

S3 Fig NGF-differentiated PC12 cells before and after exposure to oxidative stress. Cells differentiated under nerve growth factor (NGF)-supplemented were placed under the starvation medium for one day, and the CIB1-GFP-CAAX transfected cells exhibited long neurite processes before exposure to 200 μM of hydrogen peroxide. After the oxidant incubation for 2 hours, the cells presented shorter neurites and showed lower viability. Scale bar = 100 μm.
(JPG)

S4 Fig 3T3 cells with opto-Raf or opto-AKT activation showed lower cell death rates against oxidative stress. Hydrogen peroxide treatment to NIH 3T3 cells at 200 μM for 2 hours showed that the activation of opto-AKT and opto-Raf exerted protective effect compared to CIB1-GFP-CAAX control.
(JPG)

S5 Fig NGF-differentiated PC12 cells showed similar trends in preconditioning models as compared to undifferentiated PC12 cells. **(A)** Differentiated PC12 cells were illuminated with blue light for 15 minutes before they were incubated with 200 μM of hydrogen peroxide for a variable duration. The cells were kept in dark during hydrogen peroxide incubation. It was found that opto-AKT activation provided less extensive protection than opto-Raf activation for 12 hours. **(B)** Differentiated PC12 cells were illuminated with blue light for 15 minutes before they were placed in dark for varied hours of buffer period. Then, 200 μM of hydrogen peroxide was added to the culture and incubated for 2 hours under dark. Preconditioning activation of opto-Raf exhibited a delayed protective phase even after 12 hours of buffer period, while opto-AKT completely lost its protective effects after 2 hours of buffer period. For all the results, each set of data comprises of 3 sets of experiments with 1000 cells each. Data is represented as mean +/- standard deviation.
(TIF)

S6 Fig Western blot analysis of phosphorylated ERK1/2 upon blue light stimulation of cells transfected with opto-Raf system. Phosphor-ERK showed up bands upon 10 and 30 minutes of blue light stimulation, and also at 0 mins and 30 mins after being exposed to 15 minutes of blue light illumination. However, phosphor-ERK had negligible phosphorylation at 120 and 360 minutes after 15 minutes of blue light activation, suggesting that the delayed conditioning phase may be due to *de novo* protein synthesis.
(JPG)

S7 Fig Delayed phase preconditioning studies of cells with opto-Raf and singly transfected controls. 15 minutes of blue light illumination was provided to the cells before they were placed in the dark for varied hours (termed as buffer period), after which they were incubated with 200 μM of hydrogen peroxide under dark conditions for 2 hours. Opto-Raf cells exhibited two protective phases–rapid phase at the very beginning and a delayed phase with maximum protective effects at 6 hours. The five sets of data consistently showed the peak in the death rate at the 2nd hour mark of buffer period.
(JPG)

S8 Fig Postconditioning model with 2 hours of blue light illumination. 5 sets of data reveal consistently that for the AKT pathway, a 30-min delay period almost completely abolished the protective effect while there was an optimal delay period of 2 hours, which displayed similar protective effect as the set without any postconditioning delay.
(JPG)

S9 Fig Postconditioning model with 1 hour of blue light illumination. Here, a postconditioning stimulus of 1 hour is applied rather than the 2 hours applied in previous experiments. 5 sets of data reveal consistently that for the AKT pathway, a 30-min delay period almost completely abolished the protective effect. A delay period of 2 hours displayed strong protective effect, but not as strong as the set without any postconditioning delay.
(JPG)

S1 Movie Recruitment of CRY2-mCherry-Raf1 to plasma membrane marked by CIB1-GFP-CAAX in a NIH 3T3 cell. CRY2-mCherry-Raf1 was represented in the Texas Red channel in **S1 Movie** while CIB1-GFP-CAAX was shown in the green channel in **Fig 1B**. Scale bar = 20 μm
(AVI)

S2 Movie Recruitment of CRY2-mCherry-Akt to plasma membrane marked by CIB1-GFP-CAAX in a NIH 3T3 cell. CRY2-mCherry-Akt was represented in the Texas Red channel in **S2 Movie** while CIB1-GFP-CAAX was shown in the green channel in **Fig 1E**. Scale bar = 20 μm
(AVI)

S1 Table One-way ANOVA table for Figs 2 and 3 and S4 Fig. One-way ANOVA analysis was performed for Figs 2 and 3 and S4 Fig. The ANOVA tables detailed the sum of squares (SS), degree of freedom (DF) and MS (mean square) values, from which the F-statistic was calculated for each set of data and the P value was determined. Statistical significance was noted where the P value was less than 0.05, and Dunnett post-hoc test was performed for these sets of data.
(PDF)
