# Supplementary figures and images for: The Timing of Raf/ERK and AKT Activation in Protecting PC12 Cells against Oxidative Stress
Source: PLoS One. 2016 Apr 15;11(4):e0153487. doi: 10.1371/journal.pone.0153487 (PMC4833326; doi:10.1371/journal.pone.0153487)

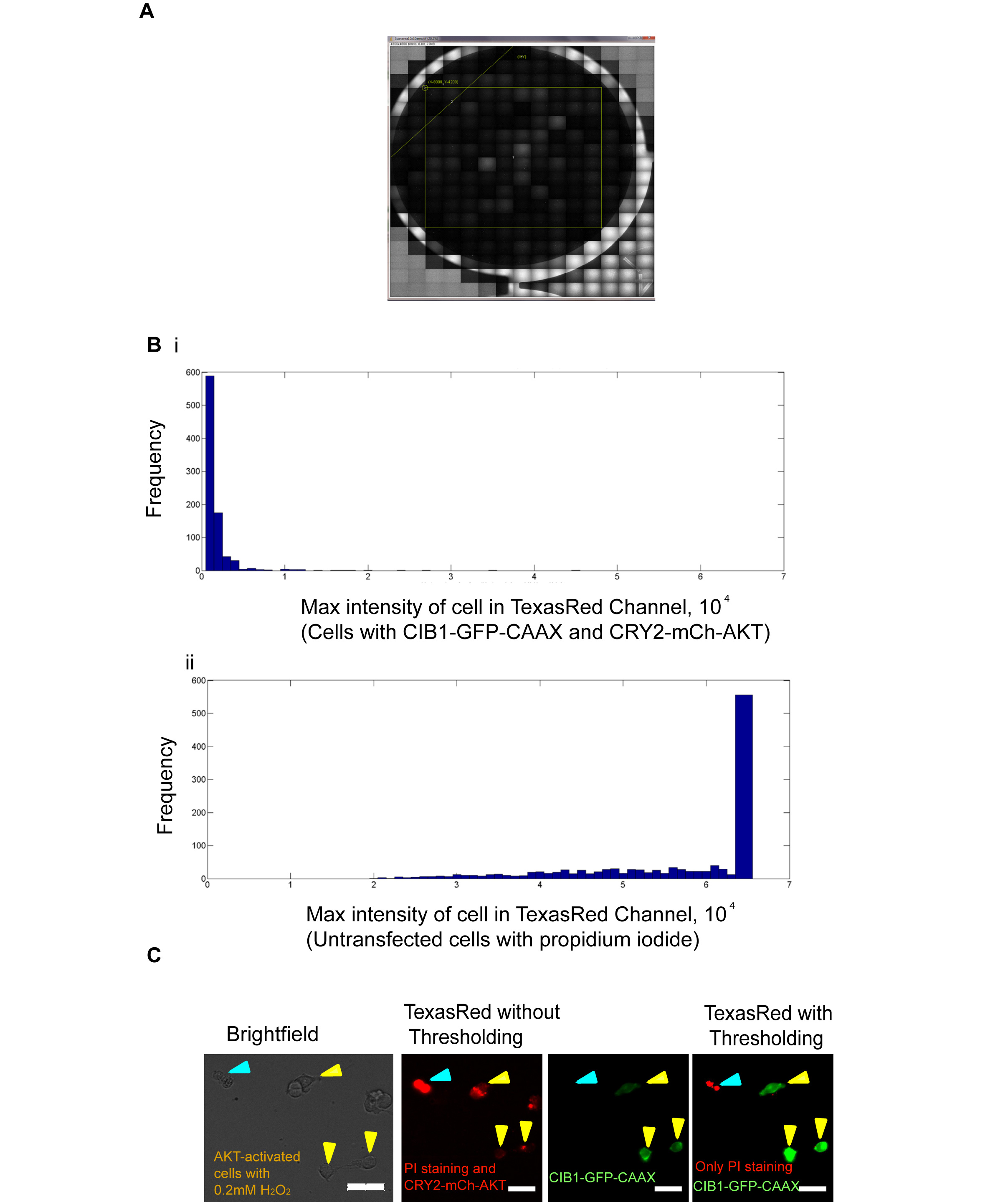

Supplement: S1 Fig — (A) Stage scan of an entire 12-well plate. 7 x 7 images were captured within the 10 x 10 area outlined for each data point. (B) Maximum fluorescence intensity of CRY2-mCherry-Akt protein was typically below 5,000, while maximum fluorescence intensity of propidium iodide was at around 60,000. This formed the basis of allowing thresholding levels, where the cut-off was imposed at 30,000. Similar intensities of light were used when capturing the images on the epifluorescence microscope for consistency. (C) The application of thresholding allowed us to differentiate between transfected dead cells and transfected cells that are alive. In this example, CRY2-mCherry-Akt and CIB1-GFP-CAAX were transfected in the cells. Scale bar = 50 μm. (JPG) [file pone.0153487.s001.jpg]

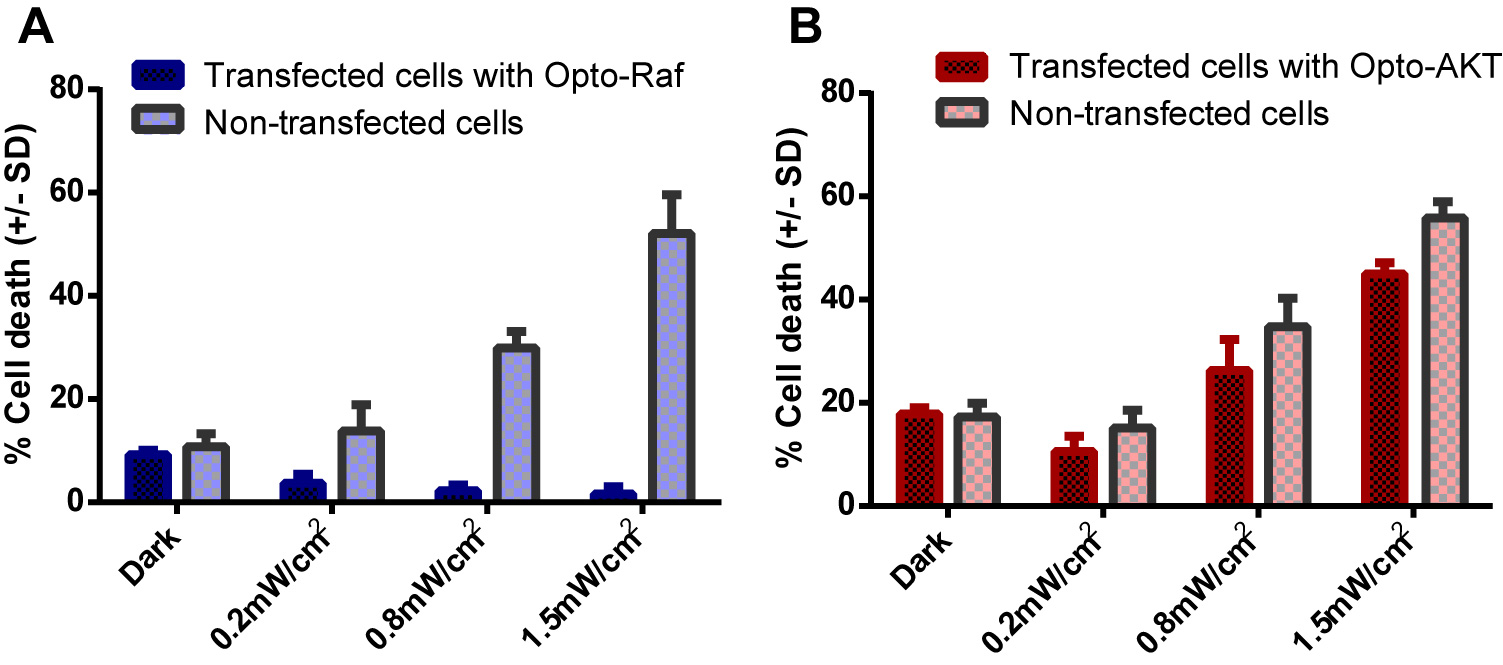

Supplement: S2 Fig — Cells were transfected with CIB1-GFP-CAAX and CRY2-mCherry, and placed under 0, 0.2, 0.8 and 1.5 mW/cm2 of blue light illumination for 24 hrs. Percentage of cell death was then probed via propidium iodide staining. The cell death rates shown in Fig 2 are comparable to the ones transfected singly with CIB1-GFP-CAAX, showing that CRY2-mCherry fragments do not affect the cell death rates significantly with/without blue light. (JPG) [file pone.0153487.s002.jpg]

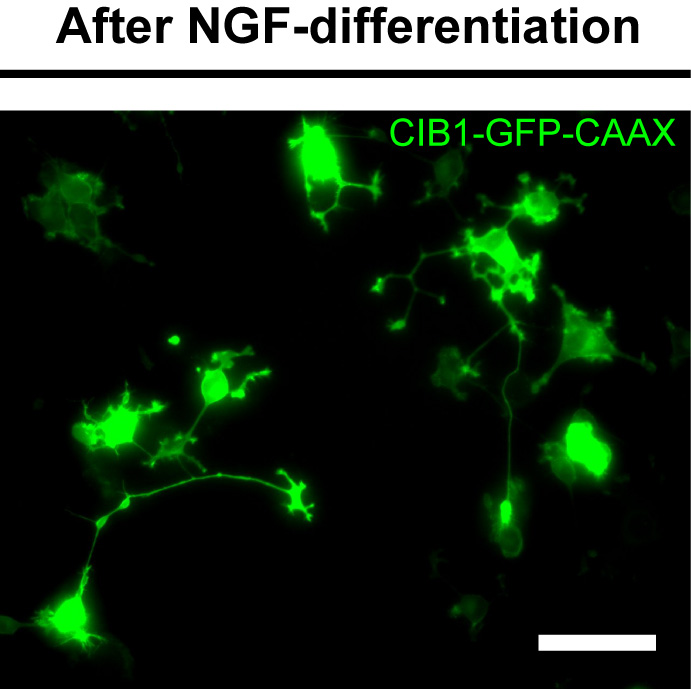

Supplement: S3 Fig — Cells differentiated under nerve growth factor (NGF)-supplemented were placed under the starvation medium for one day, and the CIB1-GFP-CAAX transfected cells exhibited long neurite processes before exposure to 200 μM of hydrogen peroxide. After the oxidant incubation for 2 hours, the cells presented shorter neurites and showed lower viability. Scale bar = 100 μm. (JPG) [file pone.0153487.s003.jpg]

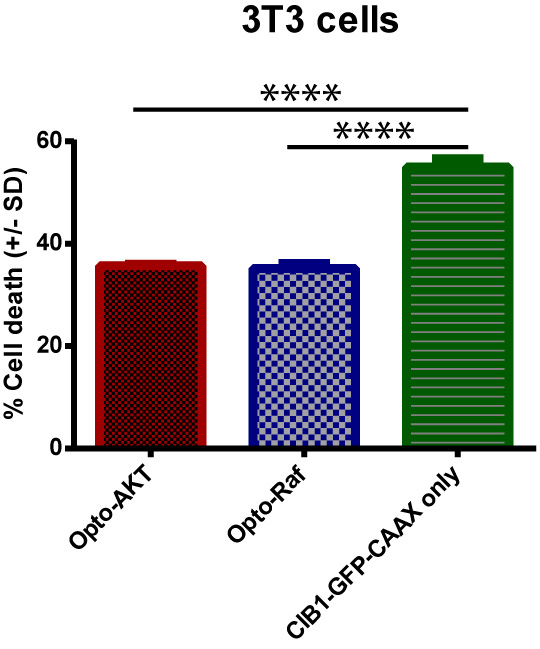

Supplement: S4 Fig — Hydrogen peroxide treatment to NIH 3T3 cells at 200 μM for 2 hours showed that the activation of opto-AKT and opto-Raf exerted protective effect compared to CIB1-GFP-CAAX control. (JPG) [file pone.0153487.s004.jpg]

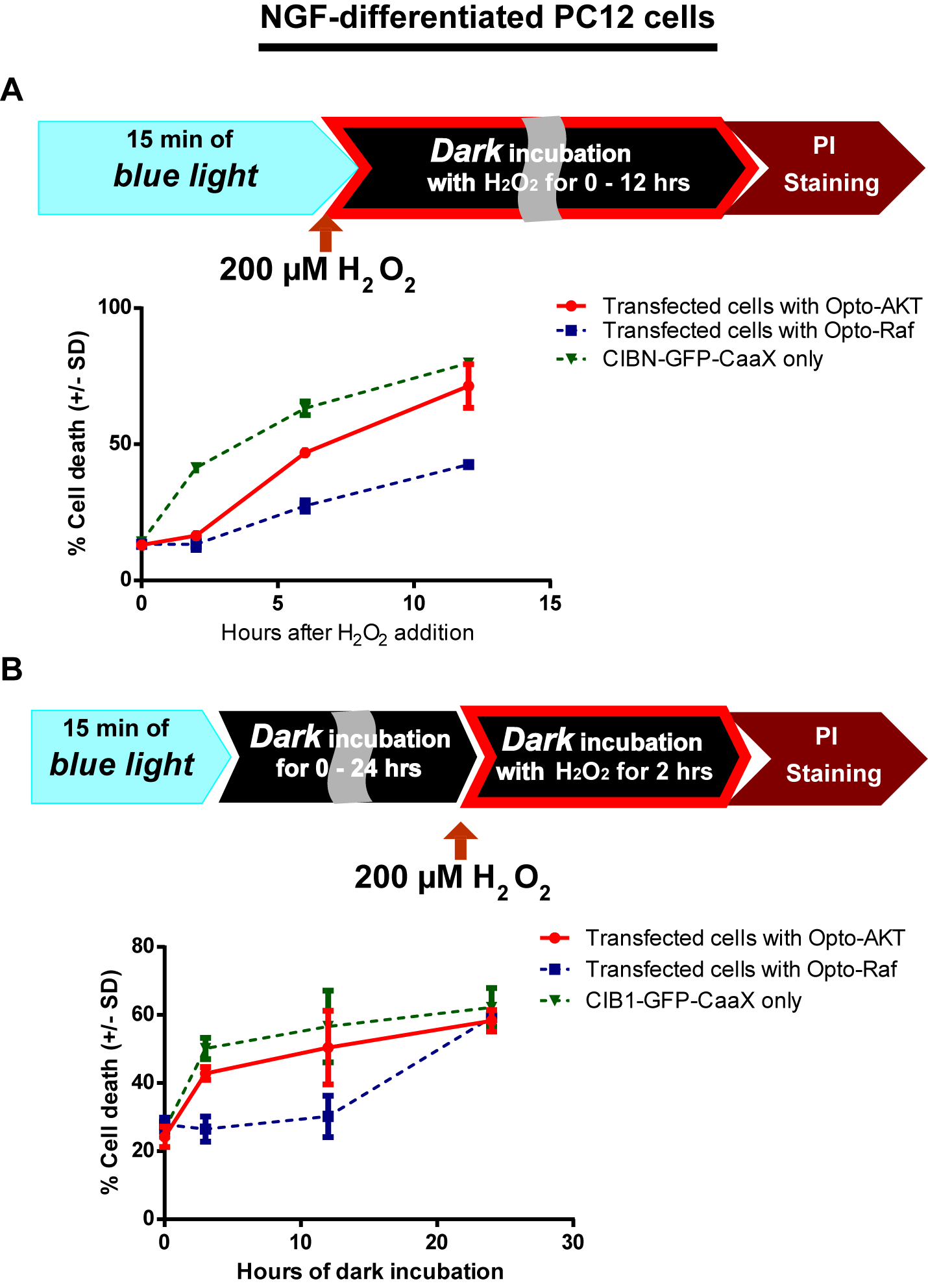

Supplement: S5 Fig — (A) Differentiated PC12 cells were illuminated with blue light for 15 minutes before they were incubated with 200 μM of hydrogen peroxide for a variable duration. The cells were kept in dark during hydrogen peroxide incubation. It was found that opto-AKT activation provided less extensive protection than opto-Raf activation for 12 hours. (B) Differentiated PC12 cells were illuminated with blue light for 15 minutes before they were placed in dark for varied hours of buffer period. Then, 200 μM of hydrogen peroxide was added to the culture and incubated for 2 hours under dark. Preconditioning activation of opto-Raf exhibited a delayed protective phase even after 12 hours of buffer period, while opto-AKT completely lost its protective effects after 2 hours of buffer period. For all the results, each set of data comprises of 3 sets of experiments with 1000 cells each. Data is represented as mean +/- standard deviation. (TIF) [file pone.0153487.s005.tif]

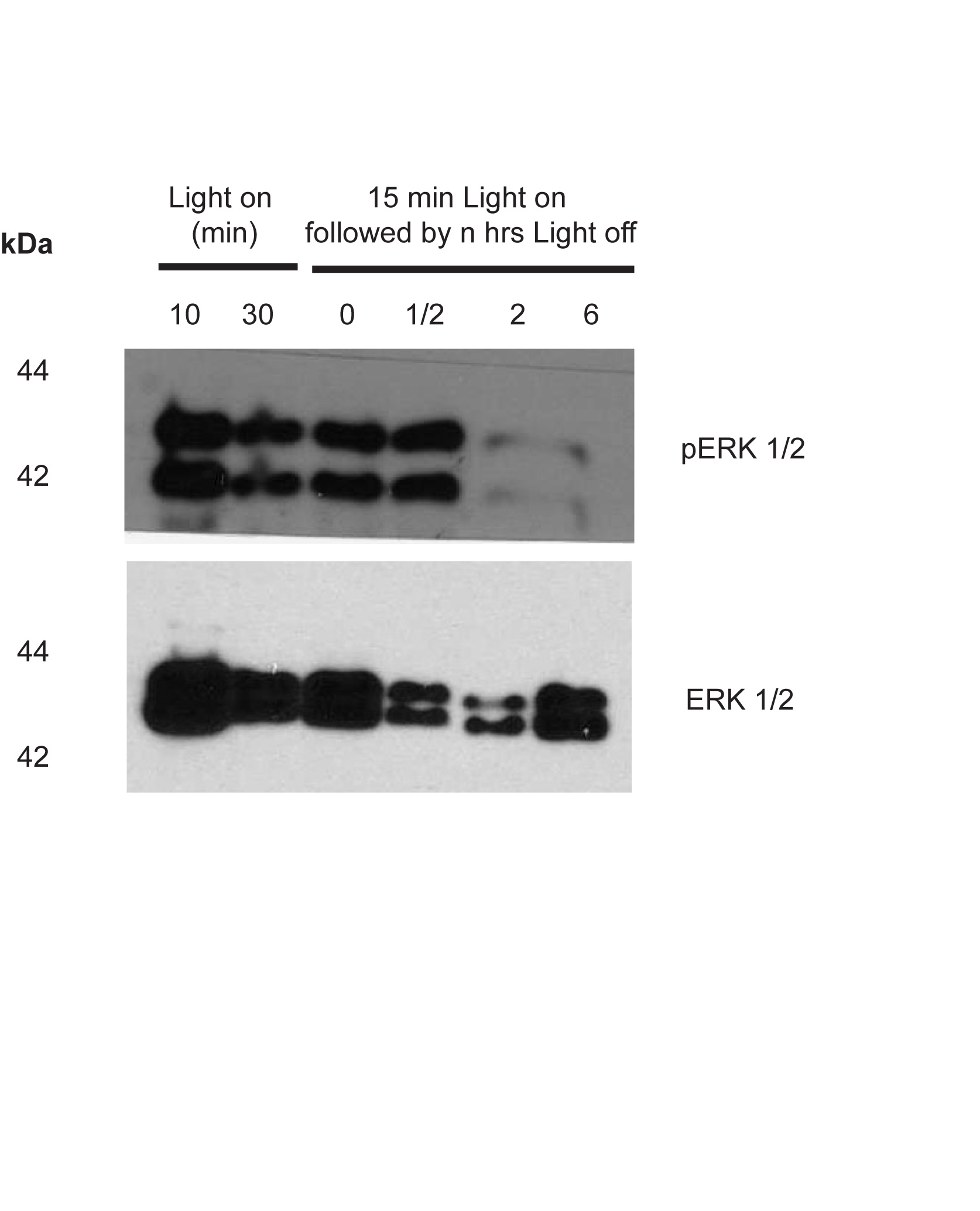

Supplement: S6 Fig — Phosphor-ERK showed up bands upon 10 and 30 minutes of blue light stimulation, and also at 0 mins and 30 mins after being exposed to 15 minutes of blue light illumination. However, phosphor-ERK had negligible phosphorylation at 120 and 360 minutes after 15 minutes of blue light activation, suggesting that the delayed conditioning phase may be due to de novo protein synthesis. (JPG) [file pone.0153487.s006.jpg]

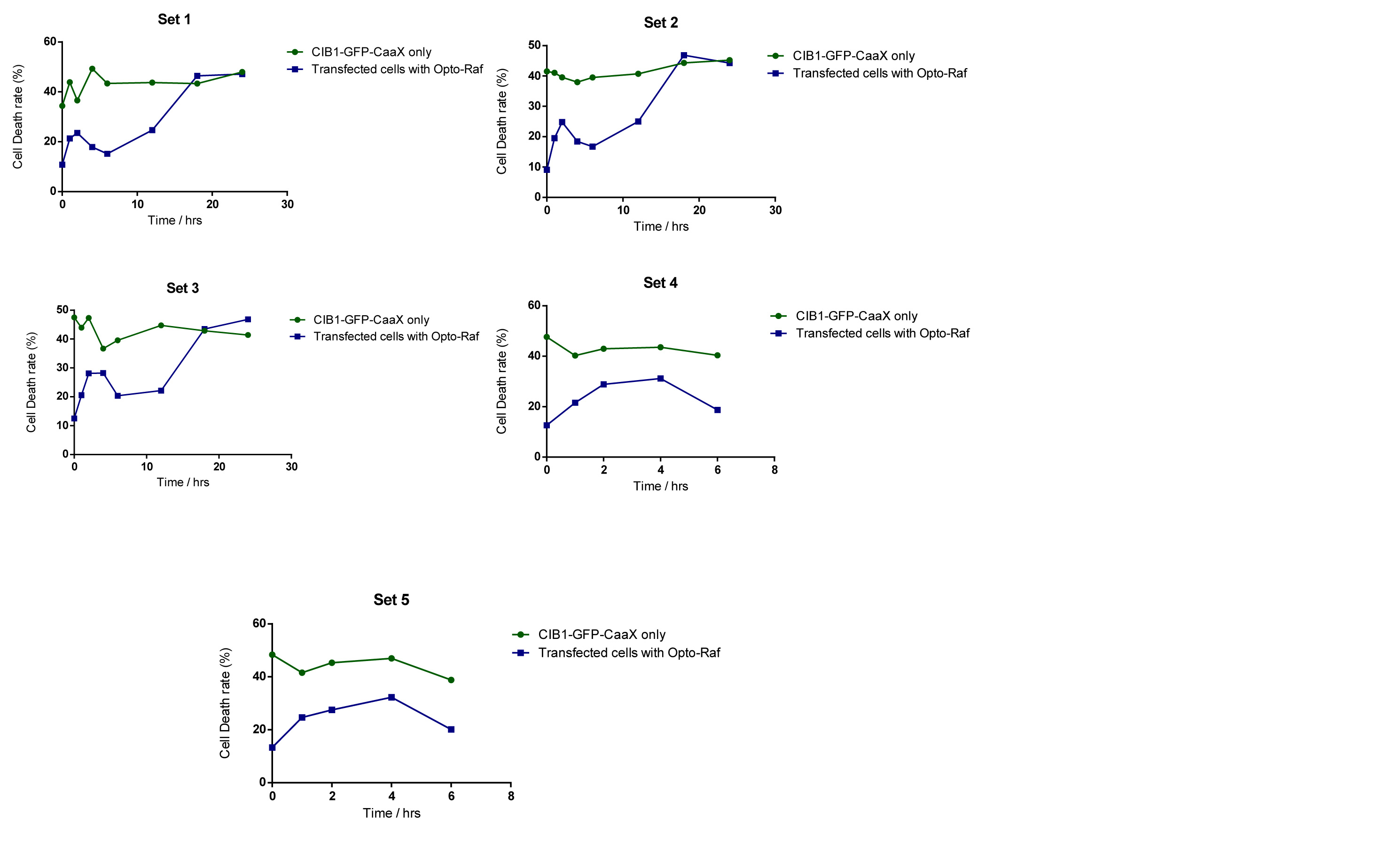

Supplement: S7 Fig — 15 minutes of blue light illumination was provided to the cells before they were placed in the dark for varied hours (termed as buffer period), after which they were incubated with 200 μM of hydrogen peroxide under dark conditions for 2 hours. Opto-Raf cells exhibited two protective phases–rapid phase at the very beginning and a delayed phase with maximum protective effects at 6 hours. The five sets of data consistently showed the peak in the death rate at the 2nd hour mark of buffer period. (JPG) [file pone.0153487.s007.jpg]

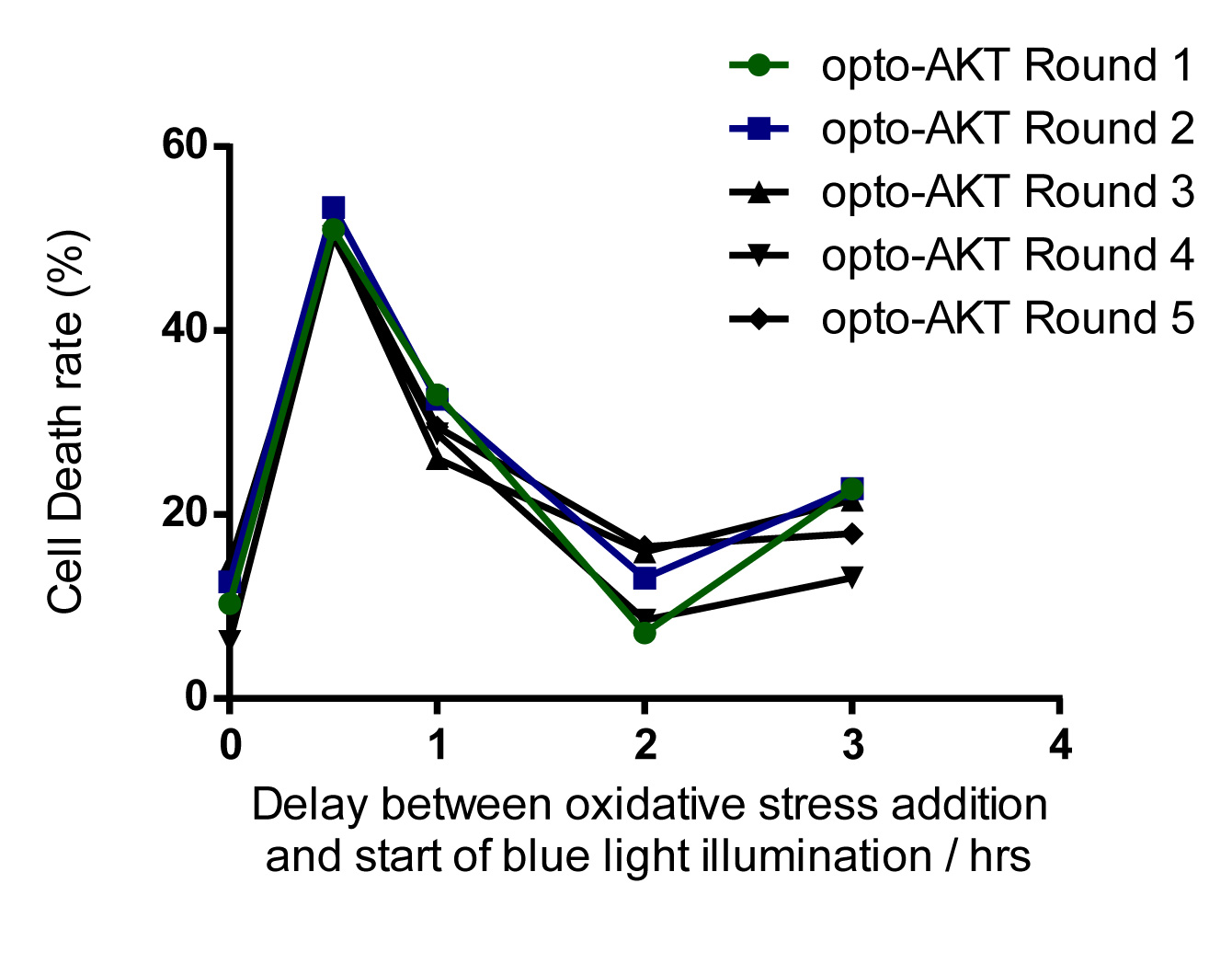

Supplement: S8 Fig — 5 sets of data reveal consistently that for the AKT pathway, a 30-min delay period almost completely abolished the protective effect while there was an optimal delay period of 2 hours, which displayed similar protective effect as the set without any postconditioning delay. (JPG) [file pone.0153487.s008.jpg]

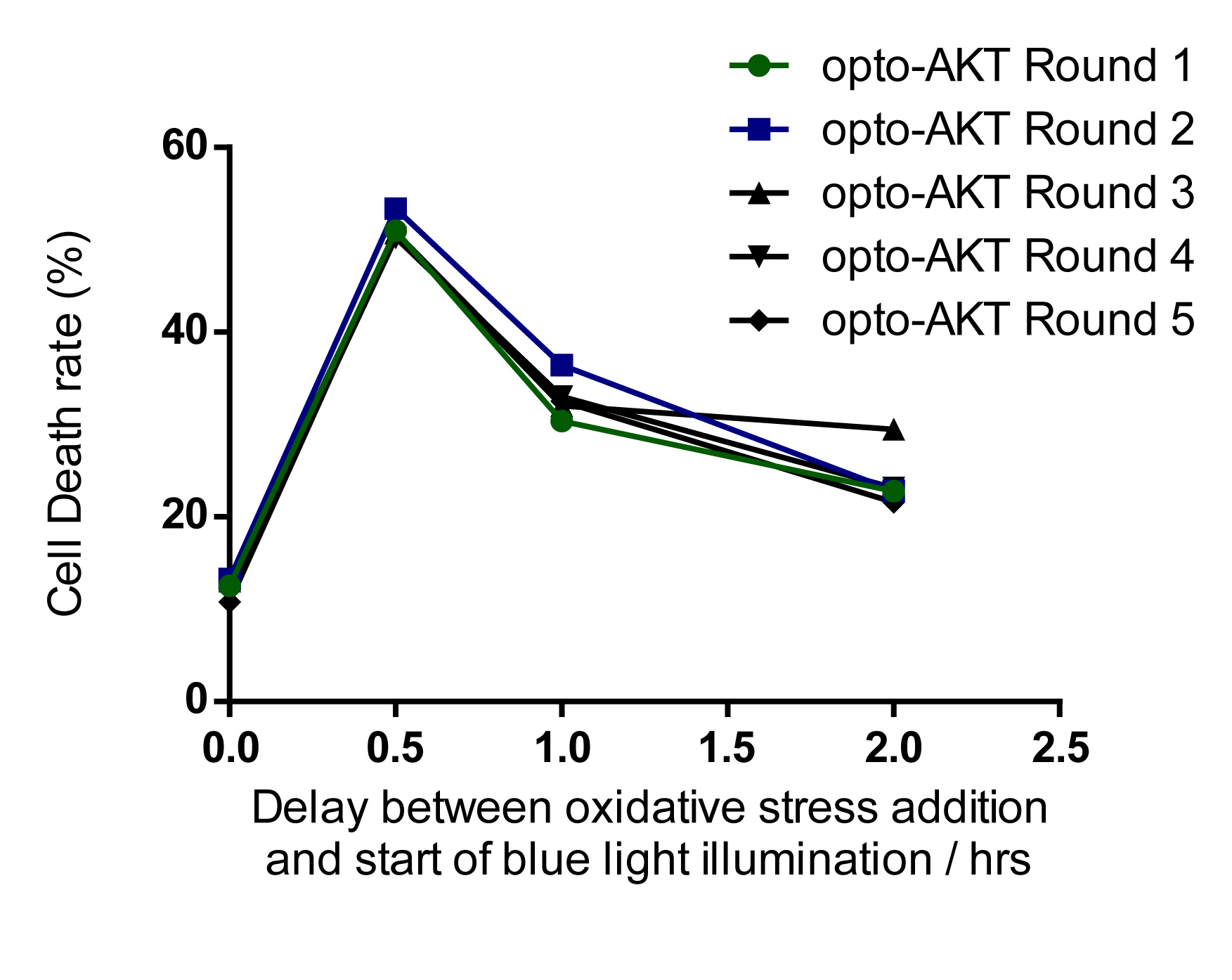

Supplement: S9 Fig — Here, a postconditioning stimulus of 1 hour is applied rather than the 2 hours applied in previous experiments. 5 sets of data reveal consistently that for the AKT pathway, a 30-min delay period almost completely abolished the protective effect. A delay period of 2 hours displayed strong protective effect, but not as strong as the set without any postconditioning delay. (JPG) [file pone.0153487.s009.jpg]
