# Supplementary material for: The Timing of Raf/ERK and AKT Activation in Protecting PC12 Cells against Oxidative Stress
Source: PLoS One. 2016 Apr 15;11(4):e0153487. doi: 10.1371/journal.pone.0153487 (PMC4833326; doi:10.1371/journal.pone.0153487)
Supplement: S1 Table — One-way ANOVA analysis was performed for Figs 2 and 3 and S4 Fig. The ANOVA tables detailed the sum of squares (SS), degree of freedom (DF) and MS (mean square) values, from which the F-statistic was calculated for each set of data and the P value was determined. Statistical significance was noted where the P value was less than 0.05, and Dunnett post-hoc test was performed for these sets of data. (PDF) [file pone.0153487.s012.pdf]

Table S1

Figure 2C

LIGHT

ANOVA table	SS	DF	MS	F (DFn, DFd)	P value
Treatment (between columns)	2414	2	1207	F (2, 6) = 316.0	P < 0.0001
Residual (within columns)	22.92	6	3.820		
Total	2437	8			

DARK

ANOVA table	SS	DF	MS	F (DFn, DFd)	P value
Treatment (between columns)	7.410	2	3.705	F (2, 6) = 0.1894	P = 0.8322
Residual (within columns)	117.4	6	19.56		
Total	124.8	8			

Figure 2D

LIGHT

ANOVA table	SS	DF	MS	F (DFn, DFd)	P value
Treatment (between columns)	1152	2	575.9	F (2, 6) = 24.31	P = 0.0013
Residual (within columns)	142.1	6	23.69		
Total	1294	8			

DARK

ANOVA table	SS	DF	MS	F (DFn, DFd)	P value
Treatment (between columns)	7.410	2	3.705	F (2, 6) = 0.1894	P = 0.8322
Residual (within columns)	117.4	6	19.56		
Total	124.8	8			

Figure 3B

LIGHT

ANOVA table	SS	DF	MS	F (DFn, DFd)	P value
Treatment (between columns)	3035	2	1517	F (2, 9) = 486.3	P < 0.0001
Residual (within columns)	28.08	9	3.120		
Total	3063	11			

DARK

ANOVA table	SS	DF	MS	F (DFn, DFd)	P value
Treatment (between columns)	66.22	2	33.11	F (2, 6) = 8.241	P = 0.0190
Residual (within columns)	24.11	6	4.018		
Total	90.33	8			

Figure 3C

LIGHT

ANOVA table	SS	DF	MS	F (DFn, DFd)	P value
Treatment (between columns)	1018	2	509.1	F (2, 12) = 39.31	P < 0.0001
Residual (within columns)	155.4	12	12.95		
Total	1174	14			

DARK

ANOVA table	SS	DF	MS	F (DFn, DFd)	P value
Treatment (between columns)	0.1053	2	0.05267	F (2, 12) = 0.005571	P = 0.9944
Residual (within columns)	113.4	12	9.454		
Total	113.5	14			

Figure 3D

LIGHT

ANOVA table	SS	DF	MS	F (DFn, DFd)	P value
Treatment (between columns)	3713	2	1856	F (2, 6) = 717.7	P < 0.0001
Residual (within columns)	15.52	6	2.587		
Total	3728	8			

DARK

ANOVA table	SS	DF	MS	F (DFn, DFd)	P value
Treatment (between columns)	3.877	2	1.938	F (2, 6) = 1.376	P = 0.3222
Residual (within columns)	8.452	6	1.409		
Total	12.33	8			

Figure 3E

LIGHT

ANOVA table	SS	DF	MS	F (DFn, DFd)	P value
Treatment (between columns)	3637	2	1819	F (2, 6) = 173.4	P < 0.0001
Residual (within columns)	62.95	6	10.49		
Total	3700	8			

DARK

ANOVA table	SS	DF	MS	F (DFn, DFd)	P value
Treatment (between columns)	66.84	2	33.42	F (2, 6) = 0.6509	P = 0.5548
Residual (within columns)	308.1	6	51.35		
Total	374.9	8			

Figure S4

ANOVA table	SS	DF	MS	F (DFn, DFd)	P value
Treatment (between columns)	765.6	2	382.8	F (2, 6) = 303.2	P < 0.0001
Residual (within columns)	7.575	6	1.263		
Total	773.1	8			

Legend:

SS: Sum of squares

DF: Degree of freedom

MS: Mean square

DFn: Degree of freedom from between the columns

DFd: Degree of freedom from within the columns

F: F-statistic
